# Supplementary material for: The evolutionary dynamics of the early Palaeozoic marine biodiversity accumulation
Source: Proc Biol Sci. 2019 Aug 28;286(1909):20191634. doi: 10.1098/rspb.2019.1634 (PMC6732384; doi:10.1098/rspb.2019.1634)
Supplement: Electronic supplementary material: The evolutionary dynamics of the Early Palaeozoic marine biodiversity accumulation [file rspb20191634supp1.docx]

**Electronic supplementary material:**

**The evolutionary dynamics of the Early Palaeozoic marine biodiversity accumulation**

Björn Kröger, Franziska Franeck and Christian M.Ø. Rasmussen

Contents:

1. Additional information on Data and Methods

2. Additional information on Results

**1. Additional information on Data and Methods**

The analysis is based on two downloads of genus level occurrence data from the Palaeobiology Database (PBDB), called here PBDB-DL#1 and PBDB-DL#2. PBDB-DL#1 was downloaded via <http://paleobiodb.org/data1.2/occs/list.txt?interval=Cambrian-Lochkovian&show=stratext,geo,loc> at 30.01.2019. PBDB-DL#2 downloaded via <http://paleobiodb.org/data1.2/occs/list.txt?show=stratext,geo,loc> at 02.02.2019.

The details of data preparation and cleaning are described in [1]. This resulted in a Paleozoic dataset with 836 families and 5985 genera from 125571 occurrences. We based our analysis on 53 stratigraphically well-defined Cambrian to Silurian time bins with an average duration of c. 2.3 myr (see [1] for details) and on stage binning’s used in the PBDB for the post Silurian data.

We estimated capture-recapture diversification and diversity dynamics by using MARK (<http://www.phidot.org/software/mark/>) through the R Package RMark version 2.2[2]. The code for RMark is available for download under https://doi.org/10.5281/zenodo.197057. The richness was calculated using the Jolly-Seber model following the POPAN formulation[3].

Diversification and diversity dynamics were estimated by using the Pradel seniority model [4]. Although this model has been developed originally for ecology, it was used for a number of palaeontological studies, e.g. [5–7], and the modelling approach has been described in much detail in these publications. In our analysis, we follow the procedure that was used also in studies by [6] [7]. We estimated survival, seniority and sampling probabilities, along with diversification rates, which are transformed into extinction (r_q_), origination(r_p_), sampling, and net diversification rates. This transformation is necessary, because probabilities between time-intervals of different lengths cannot be compared directly [6]. For a detailed description of the transformations see [6].

We calculated l_bw_ and l_fw_ as 50%, 70%, and 90%-life with the following procedure: First we used the cohorts of genera occurring in each time bin (nG_ti_) in PBDB-DL#1 as a filter to extract the post-Silurian genus occurrences from PBDB-DL#2. Based on this we created for each of the 53 cohorts a Phanerozoic presence-absence matrix with high resolution Cambro–Silurian occurrences and stage level resolution post-Silurian occurrences. These matrices were used for CR-modelling of the diversity curves of the 53 Cambrian–Silurian cohorts. Each of the resulting diversity curves had its maximum value (= nG_ti_) at the time bin of interest (t_i_). We calculated l_fw_ as difference between the maximum age of t_i_ and the minimum ages of the post-t_i_ time bins containing more than the 50%, 70% and 90% of nG_ti_. Conversely, we calculated l_bw_ as difference between the minimum age of t_i_ and the maximum ages of the pre-t_i_ time bins containing more than the 50%, 70% and 90% of nG_ti_.

In addition to this analysis we detected changepoints within the resulting time series with a test for changes in mean and variance with an approach that applies a test statistic to the entire data and determines a single changepoint based on the likelihood ratio method [8].

2**. Additional information on results**

2.1. Cambrian–Silurian richness curve

The Palaeozoic richness curve published herein is not identical with the curve published in [1]. Small differences, mostly within the level of confidence, result from each run of CR-modelling even if the original dataset remains identical. Here we publish a curve slightly different from [1] to make the differences explicit. The main difference between the curve published in [1] and our new curve is a higher early Darriwilian richness in the previously published curve. We interpret this difference as a result of the relatively poor data base for the Dapingian–early Darriwilian interval.

2.2. Cambrian–Silurian evolutionary rates

The changepoint tests for time series of individual variables resulted in single changepoints detected in the curves of the origination rates but not in the curves of the extinction rates (figure S1). The single changepoint is at the Cambro–Ordovician boundary.

In contrast, the turnover rate metrics resulting from Alroy’s [9] approach change more gradually across the Cambro–Ordovician boundary (figure S2). The metric differs in detail from the results of the CR-modelling approach (figure S2). Notably, the extinctions at the latest Katian–Hirnantian interval are less well expressed and the peak extinction metric of the LOME is during the late Katian and not during the Hirnantian as in the results from CR-modelling. Differences between the two calculations occur also in the time points of the main origination events. The origination peaks of the CE and the GOBE occur slightly earlier in the results of the CR-modelling. A detailed analysis of the causes for these differences is not in the scope of our work. Here, it is more important to emphasise on the basic similarities between the different approaches. A late Terreneuvian–early Cambrian Series 2 origination peak indicates the climax of the CE and an early–late Darriwilian origination peak indicates the climax of the GOBE in both results. The two approaches also result consistently in Cambrian extinction levels that are much higher than post-Cambrian levels.

2.3. Cambrian-Silurian survivorships

The peculiar Silurian decrease in forward survivorship (l_fw)_ and the complementary rise in l_bw_ need further discussion. We found no indication that the pattern reflects an artefact of, e.g. incomplete sampling or truncation of our data. Based on our calculations, we are confident that the decline in forward longevity through the Silurian is not an artefact, because our download PBDB-DL#2 contains the full set of early Devonian occurrences compiled in the PBDB. Furthermore, the individually checked diversity curves for the late Silurian time bin cohorts, resulting from CR-modelling are not unusual compared with the other Cambrian and Silurian curves.

This indicates that late Silurian many new genera had low chances to survive for very long into the Devonian. Indeed, a similar decline in survivorship is visible e.g. in results published in figure 2 of [10], and it probably reflects a number of late Silurian extinction events (such as the Lau, Klev and Klonk events [11, 12]).

**
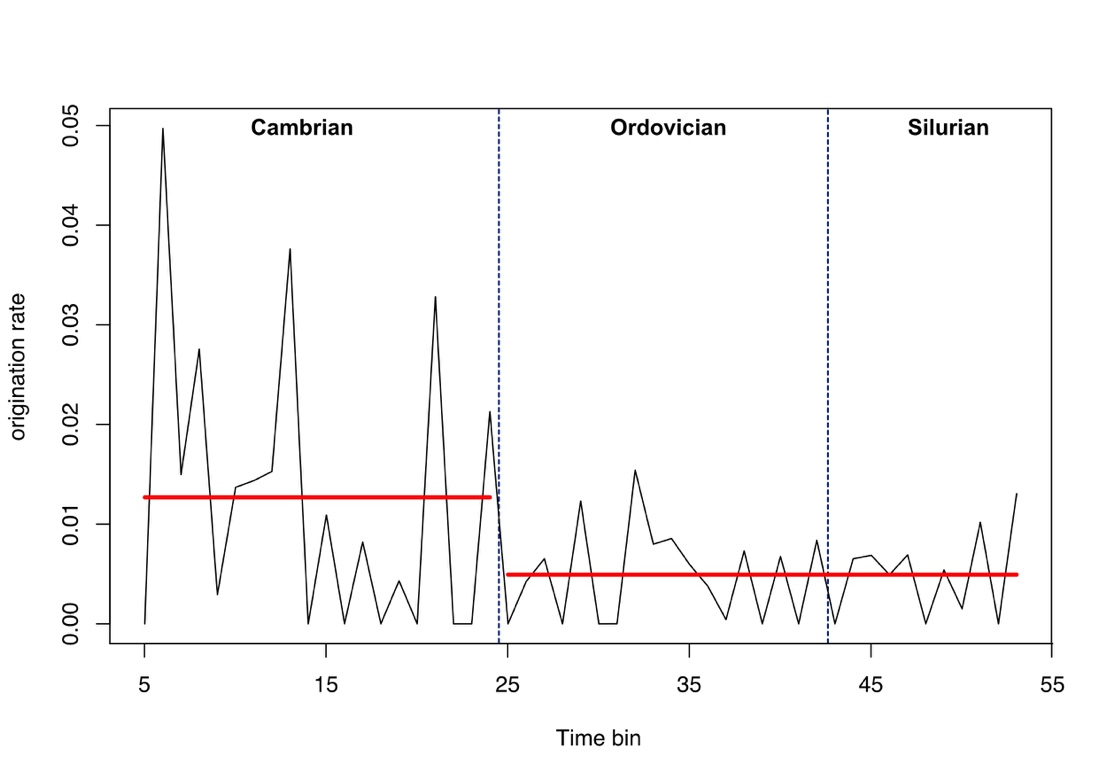
**

**Figure S1.** Time series of origination rate calculated with the CR-modelling approach. Time series starts at fifth time bin (Terreneuvian 5, see [1] for details of time bins). Horizontal bars indicate mean values before and after changepoint detected in a likelihood-based framework approach [8].


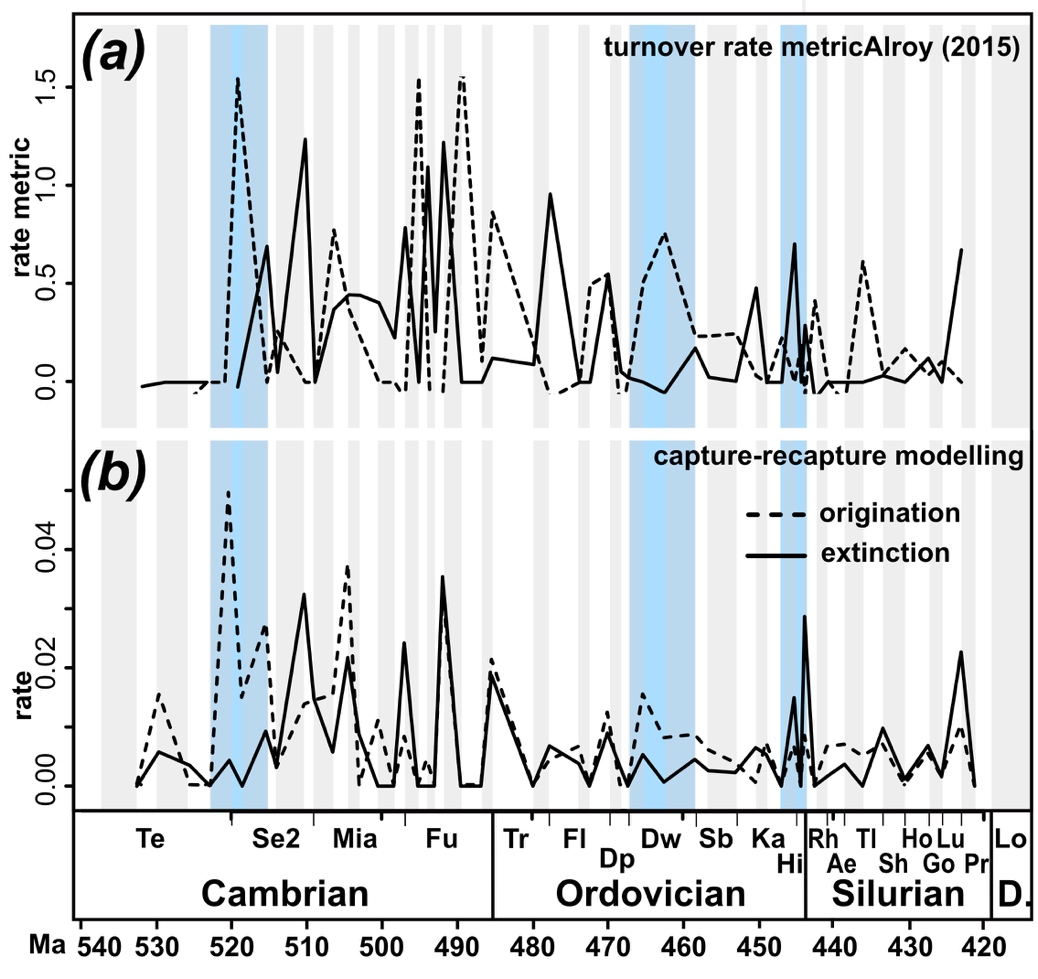


**Figure S2.** Early Palaeozoic curves of genus level origination (hatched lines) and extinction (continuous lines) calculated with the metric of Alroy [9] (a) and as rates calculated with the CR-approach (b). Abbreviations: Ae, Aeronian; CE, Cambrian Explosion; D., Devonian; Dp, Dapingian; Dw, Darriwilian; Fl, Floian; Fu, Furongian; Go, Gorstian; GOBE; Great Ordovician Biodiversification Event; Hi, Hirnantian.; Ho, Homerian; Ka, Katian; Lo, Lochkovian; Lu, Ludfordian; Mia, Miaolingian; Pr, Pridolian; Rh, Rhuddanian; Sb, Sandbian; Se2, Cambrian Series 2; Sh, Sheinwoodian; Te, Terreneuvian; Tl, Telychian; Tr, Tremadocian

**References:**

1. Rasmussen CMØ, B Kröger, ML Nielsen, J Colmenar. 2019 Cascading trend of Early Paleozoic marine radiations paused by Late Ordovician extinctions. *Proceedings of the National Academy of Sciences USA* **116**: 7207 (DOI 10.1073/pnas.1821123116).

2. Laake JL. 2013 RMark: An R interface for analysis of capture-recapture data with MARK. *AFSC Processes Rep* **2013-01**: 1-15.

3. Schwarz CJ, AN Arnason. 1996 A General Methodology for the Analysis of Capture-Recapture Experiments in Open Populations. *Biometrics* **52**: 860-873 (DOI 10.2307/2533048).

4. Pradel R. 1996 Utilization of Capture-Mark-Recapture for the Study of Recruitment and Population Growth Rate. *Biometrics* **52**: 703-709 (DOI 10.2307/2532908).

5. Connolly SR, AI Miller. 2001 Joint Estimation of Sampling and Turnover Rates from Fossil Databases: Capture-Mark-Recapture Methods Revisited. *Paleobiology* **27**: 767-751.

6. Liow LH, T Reitan, PG Harnik. 2015 Ecological interactions on macroevolutionary time scales: clams and brachiopods are more than ships that pass in the night. *Ecology Letters* **18**: 1030-1039 (DOI 10.1111/ele.12485).

7. Franeck F, LH Liow. 2019 Dissecting the paleocontinental and paleoenvironmental dynamics of the great Ordovician biodiversification. *Paleobiology* **45**: 221-234.

8. Killick R, I Eckley, A. 2014 changepoint: An R package for Changepoint Analysis. *Journal of Statistical Software* **58**: 1-19.

9. Alroy J. 2015 A more precise speciation and extinction rate estimator. *Paleobiology* **41**: 633-639.

10. Foote M, AI Miller. 2013 Determinants of early survival in marine animal genera. *Paleobiology* **39**: 171-192.

11. Calner M. 2005 Silurian carbonate platforms and extinction events—ecosystem changes exemplified from Gotland, Sweden. *Facies* **51**: 584-591.

12. Calner M. 2008 *Silurian global events--at the tipping point of climate change Mass extinction*Springer.
